# Selection of the Salt Tolerance Gene *GmSALT3* During Six Decades of Soybean Breeding in China

**DOI:** 10.3389/fpls.2021.794241

**Published:** 2021-11-16

**Authors:** Rongxia Guan, Lili Yu, Xiexiang Liu, Mingqiang Li, Ruzhen Chang, Matthew Gilliham, Lijuan Qiu

**Affiliations:** ^1^The National Key Facility for Crop Gene Resources and Genetic Improvement, Institute of Crop Sciences, Chinese Academy of Agricultural Sciences, Beijing, China; ^2^Australian Research Council Centre of Excellence in Plant Energy Biology, School of Agriculture, Food and Wine, Waite Research Institute, The University of Adelaide, Glen Osmond, SA, Australia

**Keywords:** soybean, salt tolerance, *GmSALT3*, haplotype, markers

## Abstract

Salt tolerance is an important trait that affects the growth and yield of plants growing in saline environments. The salt tolerance gene *GmSALT3* was cloned from the Chinese soybean cultivar Tiefeng 8, and its variation evaluated in Chinese wild soybeans and landraces. However, the potential role of *GmSALT3* in cultivation, and its genetic variation throughout the history of Chinese soybean breeding, remains unknown. Here we identified five haplotypes of *GmSALT3* in 279 Chinese soybean landraces using a whole genome resequencing dataset. Additionally, we developed five PCR-based functional markers: three indels and two cleaved amplified polymorphic sequences (CAPS) markers. A total of 706 Chinese soybean cultivars (released 1956–2012), and 536 modern Chinese breeding lines, were genotyped with these markers. The Chinese landraces exhibited relatively high frequencies of the haplotypes H1, H4, and H5. H1 was the predominant haplotype in both the northern region (NR) and Huanghuai region (HHR), and H5 and H4 were the major haplotypes present within the southern region (SR). In the 706 cultivars, H1, H2, and H5 were the common haplotypes, while H3 and H4 were poorly represented. Historically, H1 gradually decreased in frequency in the NR but increased in the HHR; while the salt-sensitive haplotype, H2, increased in frequency in the NR during six decades of soybean breeding. In the 536 modern breeding lines, H2 has become the most common haplotype in the NR, while H1 has remained the highest frequency haplotype in the HHR, and H5 and H1 were highest in the SR. Frequency changes resulting in geographically favored haplotypes indicates that strong selection has occurred over six decades of soybean breeding. Our molecular markers could precisely identify salt tolerant (98.9%) and sensitive (100%) accessions and could accurately trace the salt tolerance gene in soybean pedigrees. Our study, therefore, not only identified effective molecular markers for use in soybean, but also demonstrated how these markers can distinguish *GmSALT3* alleles in targeted breeding strategies for specific ecoregions.

## Introduction

Salt stress is a major environmental factor affecting agricultural plant productivity, which in turn, can threaten food security (Zhu, 2016). The ability of plants to complete their life cycles while growing in saline environments is the ultimate measure of salt tolerance (Parida and Das, 2005). Salt tolerance variation in soybean, a moderately salt-tolerant crop, has been studied throughout the last half century (Abel and MacKenzie, 1964; Fehr et al., 1971; Läuchli and Wieneke, 1979; Durand and Lacan, 1994; El-Samad and Shaddad, 1997; An et al., 2002). Salt tolerance in soybean seedlings is reportedly associated with a dominant gene proposed as *Ncl* (Abel, 1969). Additionally, the major salt tolerance quantitative trait locus (QTL) was mapped repeatedly on chromosome 3 (Chr. 3) in different salt tolerant germplasms (Lee et al., 2004; Hamwieh and Xu, 2008; Hamwieh et al., 2011; Ha et al., 2013; Guan et al., 2014a). The candidate gene *Glyma03g32900.1* has been proposed to underpin the conserved locus on Chr. 3. Researchers have isolated *Glyma03g32900.1* from different soybean germplasm, defined independently as *GmCHX1*, *GmSALT3*, and *Ncl* (Guan et al., 2014b; Qi et al., 2014; Do et al., 2016). GmSALT3 has been shown to limit Na^+^ and Cl^–^ accumulation in soybean shoots, thereby increasing soybean salt tolerance (Liu et al., 2016; Qu et al., 2021). Additionally, novel putative loci related to chloride and leaf chlorophyll concentration have been mapped on soybean chromosomes 2, 13, 14, 16, and 20, through QTL mapping and genome-wide association studies (Zeng et al., 2017; Do et al., 2018). Furthermore, Zhang et al. (2019) identified a cation diffusion facilitator, *GmCDF1* (*Glyma08g10200*), related with salt tolerance at the germination stage, but no interaction between this candidate gene and *Glyma03g32900.1* was observed. While these studies have bolstered genetic resources for breeding salt tolerant soybeans (Zeng et al., 2017; Do et al., 2018), much remains to be learned.

Genetic diversity in *GmCHX1* has been evaluated in 23 genetically distinct soybeans; a conserved coding sequence was observed in the salt-tolerant germplasm with various genotypes detected within the 12 salt-sensitive soybeans (Qi et al., 2014). Our previous research revealed nine haplotypes (H1–H9) in *GmSALT3*, with H1 observed in salt tolerant plants and H2–H5 observed in salt-sensitive soybean landraces (Guan et al., 2014b). However, little is known about the distribution of these *GmSALT3* alleles more widely across Chinese soybean cultivars.

Intriguingly, whole-genome resequencing of 106 soybeans revealed three structural variants of *GmCHX1*, of which SV-1 and SV-2, respectively, correspond to H2 and H1 of the nine Chinese soybean germplasm haplotypes, and SV-3 was a novel allele (Patil et al., 2016). Using coding regions of 216 soybean accessions from South Korea, China, and Japan, 40 haplotypes were observed, nine of which were observed in *Glycine max* accessions from China (Lee et al., 2018). Such results have encouraged us to evaluate the genomic variation of *GmSALT3* widely across representative Chinese soybean landraces, to identify novel variants, and to gain insights into how different alleles have been selected during Chinese soybean breeding over the years. Specifically, the objectives of this study were to (1) analyze genomic variation for *GmSALT3* using whole genome resequencing data of 279 representative Chinese soybean landraces, (2) develop a set of PCR-based markers and assess marker accuracy for the selection of the *GmSALT3* gene, and (3) explore how the haplotypes of *GmSALT3* have been selected and promoted in soybeans during historical breeding process.

## Materials and Methods

### Plant Materials

We obtained a diverse set of 706 soybean cultivars released during 60 years of breeding (1956–2012) from the Chinese Academy of Agricultural Sciences (CAAS), as well as 536 soybean breeding lines from the National Soybean Regional Trials in China (2013–2017). These soybeans were used to investigate the historical allelic variation in soybean salt tolerance gene *GmSALT3* and breeding lines were used to evaluate the selection efficiency of molecular markers developed from *GmSALT3*.

### Salt Tolerance Evaluation

We tested a diverse set of 536 soybean breeding lines for salt tolerance in a rain shelter that excluded rainfall but allowed plants to grow under ambient light and temperature (Institute of Crop Sciences, CAAS). Experiments were performed according to previous reports (Jiang et al., 2013; Liu et al., 2016). Ten seeds of each line were sowed in a pot filled with vermiculite and were thinned to six plants after five days. Twenty-four pots were placed in one tray and 11 days after sowing (DAS), when the unifoliate leaves were fully expanded, 2 L of salt solution (200 mM NaCl) was added to each tray. The same volume of NaCl solution was added to each tray at 13 and 15 DAS, respectively. Ten days after the last addition of the salt solution, leaf chlorosis was observed and scored accordingly: 1 = healthy green leaves, no damage observed; 2 = slight chlorosis, light yellowish color observed in true leaves; 3 = moderate chlorosis, chlorosis observed in trifoliate leaves; 4 = severe chlorosis, more than 75% of the leaf area showed chlorosis; 5 = dead, plants were completely withered. The experiment was performed with 3 pots of each genotype.

### Analysis of Genomic Sequence Variation

We used the genomic dataset of 279 Chinese soybean landraces (Supplementary Table 1; Li et al., 2020) to determine genomic sequence variation in *GmSALT3.* SNPs with a minor allele frequency ≤0.01, or with missing data >0.1, and indels with maximum length >10 bp were discarded. Annotation was carried out based on the soybean reference genome Wm82.a2.v1^1^ and transcript sequence information (Guan et al., 2014b; Qi et al., 2014; Lee et al., 2018). SNPs and indels within the 5′UTR and the genomic region of *GmSALT3* were used for haplotype investigation.

### Development of Functional Markers

We developed functional markers to distinguish the haplotypes of *GmSALT3*. We created three indel markers based on the ∼150 bp insertion in the promoter region, the 4 bp deletion and the 3.78-kb insertion in the coding sequence of *GmSALT3*. Additionally, we developed cleaved amplified polymorphic sequences (CAPS) markers, H3-*Mbo*II and H4-*Nla*III, for haplotypes H3 and H4 based on a GC > TG substitution at exon 4 and a splice site AG > AT substitution at the end of intron 2. The primers used to amplify and distinguish haplotypes of *GmSALT3* are shown in Table 1.

**TABLE 1:** Haplotype-specific markers for *GmSALT3*.

Marker name		Primer sequence	Marker type	Size (bp)
Pro-Ins	F	GGGTTGTGCCTAAATAGCA	Indel	623/775/777
	R	AAGGAAGAGCGTGGTTCA		
H2-Ins	F	GCGGGAGTAATGTTATCGG	Indel	
	R-H1	CGATTAGCTCCACCAACCCT		364
	R-H2	GTCGTATCTTGGGAGAGGAG		565
H3-*Mbo*II	F	TATGGTGGCTAAGCAGGTG	CAPS	(125 + 119 + 52)/(244 + 52)
	R	CAGTGAGTTCGGTAAGTTGC		
H4-*Nla*III	F	AAAGCGCATAAGTTATAACACAAAAT	CAPS	(133 + 129)/(111 + 87 + 42 + 22)/(133 + 87 + 42)
	R	GAATGTAACCCTATCATGTCTGTCA		
H5-Del	F	CTGTCCATCACGGCTTTCC	Indel	210/214
	R	CTATAGTAGGTCCACCTGAGAA		

### Genomic DNA Isolation and Genotyping of Soybeans

Genomic DNA was isolated from leaves of each accession by using a Genomic DNA Purification Kit (Thermo Fisher Scientific, Lithuania), and 100 ng DNA was used for PCR amplification with a T100 thermal cycler (Bio-Rad). We performed the PCR experiment using 20 μl reaction mixtures containing 100 ng genomic DNA, 2 μl 10× EasyTaq Buffer (with Mg^2+^), 1.5 μl 2.5 mM dNTPs, 2 μl each of 2 μM primer stock, and 1 U EasyTaq DNA Polymerase (TransGen Biotech, Beijing, China) under the following thermal cycler conditions: 95°C for 5 min, then 35 cycles at 95°C for 30 s, 55°C for 30 s, and 72°C for 50 s, and followed by a final extension of 5 min at 72°C. Products of PCR of H3-*Mbo*II and H4-*Nla*III were digested with, respectively, 1 U of restriction enzymes *Mbo*II and *Nla*III in 1× reaction buffer for 1 h at 37°C. PCR products and enzyme-digested products were separated on a 1.5% agarose gel or 6% polyacrylamide gel.

## Results

### Haplotypes of *GmSALT3* and Their Geographical Distribution

**FIGURE 1:**
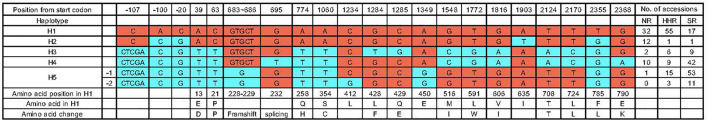
Haplotypes of *GmSALT3*. Red color represents the same nucleotides as that of H1 type, blue color represents variation in the 5′UTR and coding regions different from that of H1 type. See Supplementary Table 2 for the whole genomic variation of *GmSALT3*.

The multi-allelic salt tolerance gene *GmSALT3*, was previously isolated from Chinese soybean cultivar Tiefeng 8, and five distinct haplotypes (H1–H5) have previously been described in Chinese soybean landraces (Guan et al., 2014b). H1 was found in the salt tolerant soybean Tiefeng 8. H2 was found in salt sensitive soybean cultivar 85-140, of which a 3.78-kb retro transposon insertion resulted in a truncated GmSALT3 protein (reference Williams 82: W82). Compared with the H1 haplotype, H3–H5 had a ∼150 bp insertion at position-147 of the promoter region and a TCGA insertion at position-103 in common with each other (Guan et al., 2014b). We analyzed the haplotypes of *GmSALT3* from a panel of 279 Chinese landraces (Supplementary Table 1) and identified 57 polymorphic sites, including 53 SNPs and four indels (Supplementary Table 2). These SNPs and indels formed six haplotypes (H1–H4, H5-1, and H5-2) (Figure 1 and Supplementary Table 2). H1–H4 were the same as reported previously (Guan et al., 2014b). Both H5-1 and H5-2 had a 4 bp deletion in exon 2 which resulted in frameshift as earlier reported in H5, while H5-1 differed from H5-2 by 10 SNPs and one indel in intron 1 (Supplementary Table 2). Given that H5-1 and H5-2 had identical 714-nt cDNA sequence, we ascribed them as haplotype H5.

We found that H1, H4, and H5 were the highest frequency haplotypes in Chinese soybean landraces (37.3, 21.8, and 29.7%, respectively). When examining the three main eco-regions, the northern region (NR) which includes the area above 40°N, the Huanghuai region (HHR) in middle China (30°N to 40°N), and the southern region (SR) which ranges from Hainan Island (19°N) to Shanghai (31°N) (Li et al., 2008), H1 was the most abundant haplotype in both the NR and the HHR, while H4 and H5 together, were the most abundant haplotypes in the SR of China (Figure 1, Supplementary Figure 1, and Supplementary Table 1).

### Development of Haplotype-Specific Markers

**FIGURE 2:**
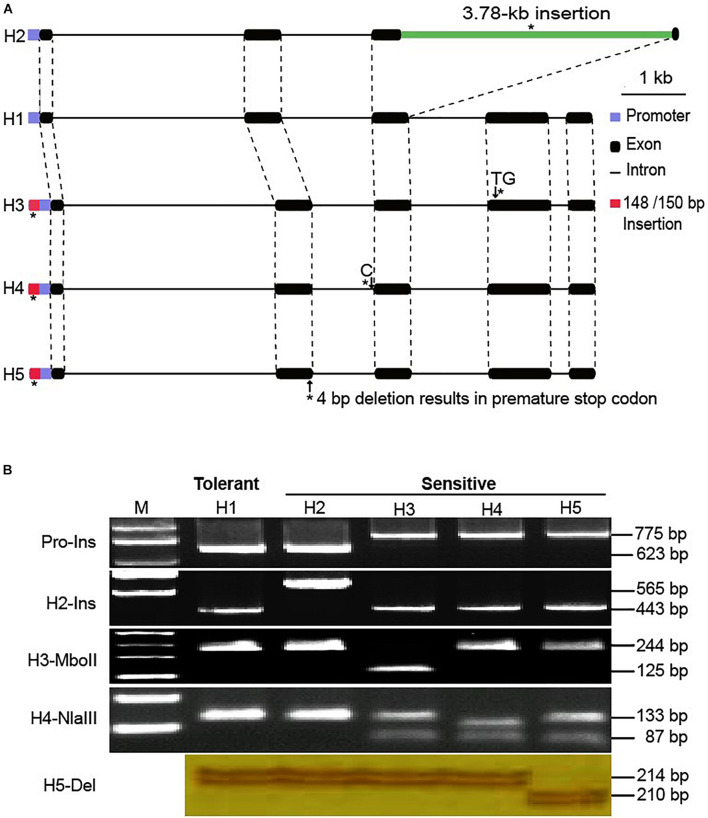
Variation of *GmSALT3* and haplotypes (H1–H5) revealed by indel and CAPS markers. Schematic diagrams show the structure of five *GmSALT3* haplotypes. Asterisks indicate variations that were used for PCR-based marker development **(A)**; Markers that distinguish five haplotypes in soybean varieties **(B)**.

To identify different *GmSALT3* alleles in a simple and effective way, we developed three indel markers and two CAPS markers. Indel marker Pro-Ins was designed to distinguish H1 and H2 haplotypes from H3 to H5; the H1 and H2 haplotypes amplified a 623 bp fragment, while H3–H5 amplified an approximate 775 bp fragment due to ∼150 bp insertion in the promoter region (Figure 2). Indel marker H2-Ins was designed to distinguish H2 from the other haplotypes using a common forward primer (H2-Ins-F) and two haplotype-specific reverse primers that were designed according to a 3.78-kb insertion (H2-Ins-R/H2) or exon 3 (H2-Ins-R/H1), and amplified a 565 bp fragment in H2, and 364 bp fragment in the H1 and H3–H5 haplotypes (Figure 2B). The marker H5-Del amplified a 210 bp sequence from H5 and a 214 bp sequence from the other four haplotypes. The CAPS marker H3-*Mbo*II amplified a 296 bp fragment from all haplotypes, and the fragment from the H3 haplotype could be digested into three fragments (125, 119, and 52 bp) by *Mbo*II, whereas the products of the other haplotypes were cleaved into two fragments (244 and 52 bp). To distinguish H4 from the other four haplotypes, CAPS marker H4-*Nla*III was developed, and an amplicon of H4 was digested into four fragments (111, 87, 42, and 22 bp) by *Nla*III, the products of H1 and H2 were digested into two fragments (133 and 129 bp) and those of H3 and H5 into three fragments (133, 87, and 42 bp) (Figure 2B).

### Changes of *GmSALT3* Haplotype Frequencies During Six Decades of Chinese Soybean Breeding

**FIGURE 3:**
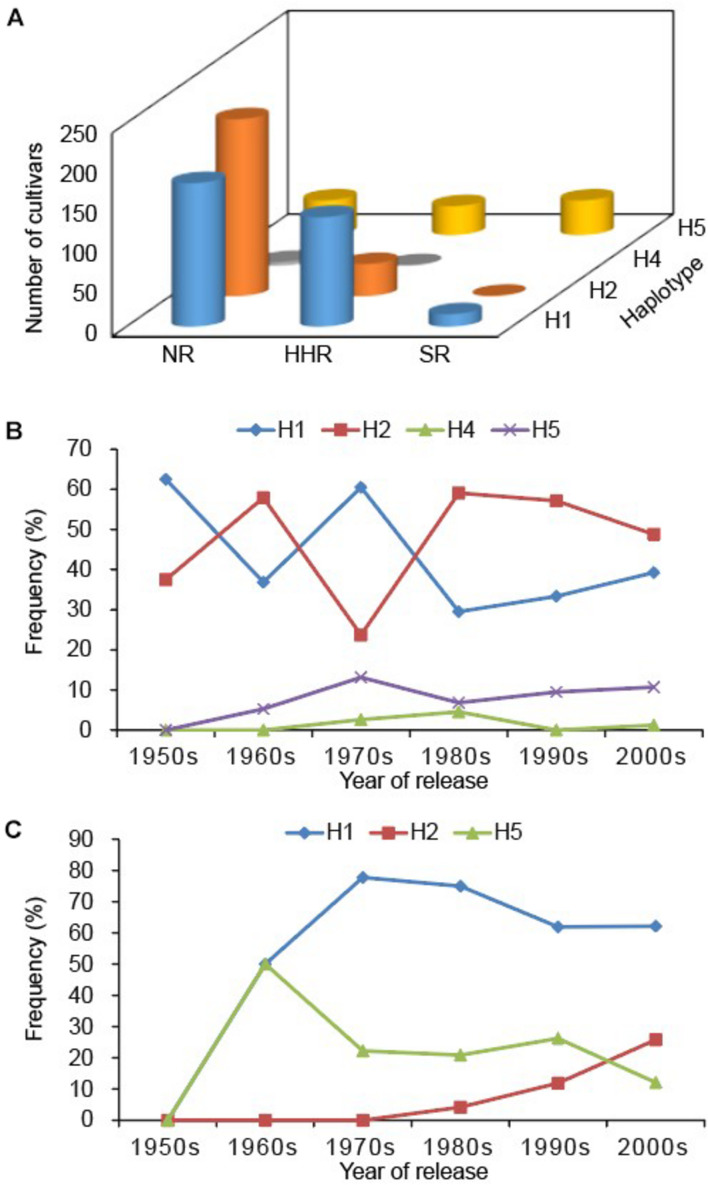
*GmSALT3* allele characterization in Chinese soybean varieties. Distribution of haplotypes among varieties from different ecoregions **(A)**; Frequency changes of haplotypes in the northern region of China (NR) **(B)**; and Huanghuai region of China (HHR) **(C)** over 60 years of soybean breeding. The distribution of H3 is not shown because only five cultivars expressed this haplotype.

We used the markers developed in this study to explore the genetic diversity of *GmSALT3* in a total of 706 modern Chinese soybean cultivars (released between 1956 and 2012). All five haplotypes were found in these cultivars (*n* = 323 H1, 259 H2, 5 H3, 6 H4, and 111 H5). Different haplotypes were not equally distributed throughout the three eco-regions in China. The frequencies of cultivar haplotypes in the NR were as follows: 39.6% H1, 49.1% H2, 0.2% H3, 1.0% H4, and 8.7% H5. In the HHR, 63.1% of the genotypes were H1, 18.7% were H2, 0.9% were H3, 0.4% were H4, and 16.8% were H5. Intriguingly, H5 was the most frequent haplotype (68.8%) in the SR, followed by H1 (25.0%), H3 (2.1%), and H4 (4.2%); H2 was the least frequent haplotype, found in only one cultivar (Figure 3A).

Additionally, we analyzed frequency changes of the haplotypes in the NR and HHR since the 1950s, at 10-year release intervals, over a 60-year time span [SR was not included due to a smaller sample of cultivars (48)]. Eight cultivars released in 2011 and 2012 were merged with those of the 2000s. In the NR, H1 and H2 frequencies exhibited fluctuating patterns from the 1950s to 1970s (Figure 3B). H1 decreased from 60.5% to 39.3% and H2 increased from 23.7 to 48.8% during the 1970s to 2000s (Figure 3B). H3 and H4 occurred at low frequencies, where they were, respectively, present in only 1 and 6 cultivars.

**FIGURE 4:**
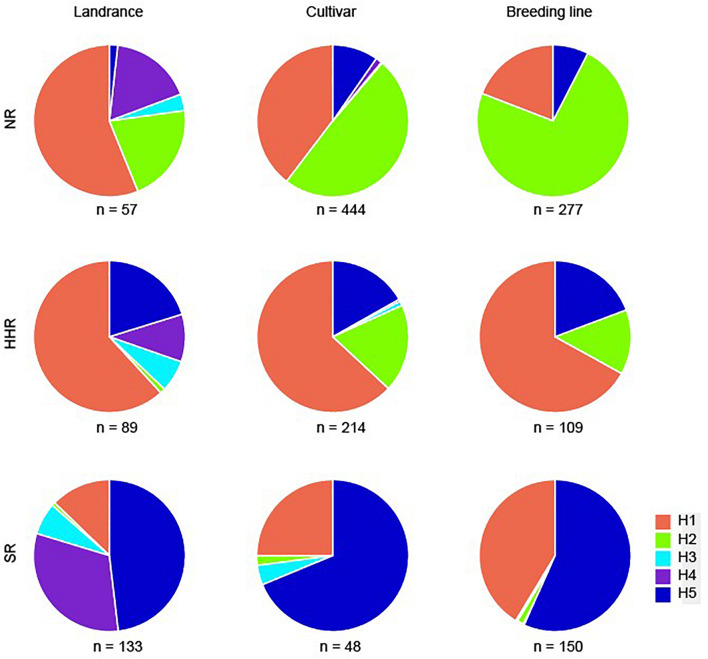
Distribution of *GmSALT3* haplotypes in Chinese soybean landraces, cultivars, and breeding lines from the three ecoregions. NR, northern region of China; HHR, Huanghuai region of China; SR, southern region of China.

Five haplotypes were observed in the HHR; where H1 increased in frequency from 50.0 to 77.8% (1960s to 1970s), then gradually decreased to 62.5% in the 21^st^ century. H2 increased in frequency to 25.8% and H5 decreased to 12.1% (Figure 3C). Such variation in allelic frequency over the past 60 years indicates that H1 and H2 were selected for in the NR, and H1 was favored in the HHR (Figure 4).

### Validation of Functional Markers in Modern Soybean Breeding Lines

To evaluate the genotype-phenotype relationship among haplotypes in modern soybean lines, a validation panel of 536 breeding lines (National Soybean Regional Trials, 2013–2017) was tested for salt tolerance and genotyped using the five PCR-based functional markers determined in this study. Four haplotypes were observed in the breeding lines (Figure 4), with 35.1% of soybean lines possessing H1, 41.0% possessing H2, and 23.7% possessing H5. H3 was only observed in one line from the SR. In the NR, H2 was the predominant haplotype (73.3%); H5 was only observed in 7.6% of lines. Whereas in the HHR, H1 and H5 were expressed in 67.0% and 19.3% of lines, respectively. In the SR, H5 and H1 were the two highest frequency haplotypes (56.7% and 41.3%). Modern breeding line frequencies of H2 in the NR, and H1 in the SR, were much higher compared with soybean cultivars released from 1956 to 2012 (Figure 4). These results suggest positive selection for favored haplotypes in different eco-regions during modern cultivation.

**FIGURE 5:**
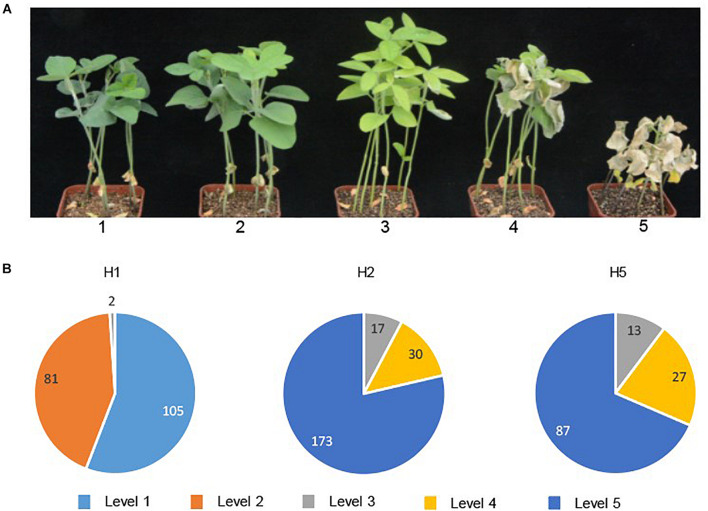
Salt tolerance evaluation of 536 modern soybean breeding lines. Chlorosis scores were used for salt tolerance rating after 15 days of NaCl treatment (plants with score ratings 1 or 2 were considered salt tolerant; 3–5 were salt sensitive) **(A)**; In each haplotype, the number of lines belonging to each chlorosis score for salt tolerance ratings (level 1 to level 5) are shown **(B)**.

Of the 536 modern breeding lines, 186 lines were salt tolerant (with chlorosis scores of 1 or 2) and 350 lines were salt sensitive (with chlorosis scores 3–5) (Figure 5). The correlation of genotype-phenotype showed that H2 (220 lines), H5 (127 lines) and one breeding line which possessed H3, were all salt sensitive, while 98.9% of the lines possessing H1 were salt tolerant (Figure 5B). Two breeding lines with the H1 haplotype showed moderate leaf scorch (chlorosis score 3), suggesting that different loci other than *GmSALT3* might affect salt tolerance in these accessions.

### Pedigree Tracing of *GmSALT3* in Soybean Varieties

**FIGURE 6:**
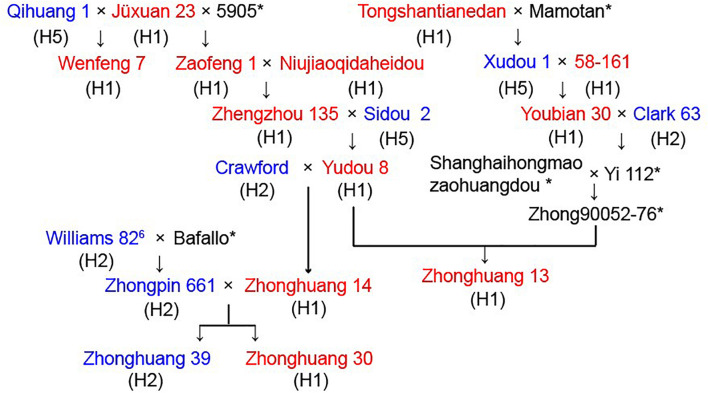
Pedigree of four soybean cultivars for the salt tolerance gene *GmSALT3*. Salt tolerance of each cultivar is indicated in red (tolerant), blue (sensitive), and haplotypes (H1–H5) are shown in parentheses under the name of cultivar. * indicate seeds of these cultivars were unavailable.

To examine potential use of the *GmSALT3* PCR-based markers in a soybean breeding program, we traced the pedigree of four soybean cultivars. Zhonghuang 13 is a salt tolerant cultivar that exemplifies widely adaptable soybean cultivar in China, it was registered in nine provinces ranged from 29°N to 42°N, and was also the first Chinese soybean cultivar used for *de novo genome* assembly (Shen et al., 2018). Wenfeng 7 is a historical soybean cultivar that has previously been used for salt tolerance analysis (Ren et al., 2012; Pi et al., 2016), and shares Jüxuan 23 as the common ancestral parent with Zhonghuang 13 (Figure 6). Zhonghuang 30 is an abiotic stress resistant cultivar that is widely used in the NR (He et al., 2016), and Zhonghuang 39 is a salt sensitive sister line of Zhonghuang 30. These cultivars and 19 related ancestral lines in the pedigree were assayed with the five molecular markers. The phenotypic salt response and corresponding *GmSALT3* haplotypes (H1–H5) of the pedigree lines are marked in Figure 6. Zhonghuang 13, Zhonghuang 30, Wenfeng 7 were H1 type salt tolerant cultivars. The salt tolerance gene in Wenfeng 7 could be traced back to Jüxuan 23. The tolerance gene in Zhonghuang 13 could be traced back to Zhengzhou 135 or 58-161. The *GmSALT3* allele (haplotype H2) in Zhonghuang 39, the sensitive sister line of Zhonghuang 30, was traced back to W82, while the tolerant allele in Zhonghuang 30 was inherited from Zhengzhou 135 (Figure 6). These results indicate that the H1 haplotype is present in all the salt tolerant ancestors in the pedigree, and that sensitive ancestors expressed haplotypes H2 or H5.

## Discussion

### Genomic Diversity of *GmSALT3* in Chinese Soybean Landraces

Gene cloning related to agronomic traits has facilitated the development allele-specific functional DNA markers, which in turn, have been used in soybean research to validate genotype-phenotype relationships and facilitate breeding efforts (Shin and Lee, 2012; Tsubokura et al., 2013, 2014; Jia et al., 2014; Zhai et al., 2014; Liu et al., 2015). *GmSALT3* was identified as gene that controls Na^+^ and Cl^–^ uptake in soybean shoots and improves yield in saline conditions without negative consequences on soybean yield under non-saline field conditions (Liu et al., 2016). We previously found at least nine haplotypes of *GmSALT3* in 172 Chinese soybean landraces and 57 wild soybeans by analyzing promoter and coding sequences of the gene (Guan et al., 2014b). Additionally, 3 and 40 different haplotypes of *GmSALT3* were observed in 106 and 216 diverse soybean accessions, respectively (Patil et al., 2016; Lee et al., 2018). In the present study, genetic diversity in *GmSALT3* was analyzed using whole genome resequencing data from 279 representative Chinese soybean landraces (Li et al., 2020). Genomic variants were classified into six haplotypes (H1–H4, H5-1, and H5-2). Haplotypes H1–H4 were the same as previously found. The intron variations between Haplotype H5-2 and H5-1 (Supplementary Table 2), indicate that the common frameshift mutation (TGCT deletion) in exon 2 of H5-1 and H5-2 arose independently in south of China from different lineages (Figure 1 and Supplementary Figure 2). H5-1 and H5-2 were assigned as H5 haplotype due to the same frameshift mutation and their identical amino acid sequences (Figure 1).

In the 40 *GmSALT3* haplotypes observed by Lee et al. (2018), Ha, HTn, Hd-2, and Hd-3, which correspond to H1, H2, H5-1, and H5-2 in present study, were the four majority haplotypes in cultivated soybean (Lee et al., 2018). Haplotypes Hst-7, Hss-11, Hss-12, and Hd-1 were shown in only one Chinese soybean accession in their report, indicating these are minor alleles in Chinese soybeans. The H3 and H4 haplotypes identified in the present study were not found in any of the three structural variations listed in Patil’s report (Patil et al., 2016), or in the 40 haplotypes of Lee’s study (Lee et al., 2018). This lack of concordance is not surprising considering the greater number of Chinese soybean accessions used in the present experiment.

### Functional Markers of *GmSALT3* and Their Potential Use in Soybean Breeding

Using the functional markers we developed in the present study (Figure 2 and Table 1), we successfully validated the predictive accuracy of the markers across 536 modern breeding lines. Prediction accuracy was 98.9% for salt-tolerant lines and 100% for salt-sensitive lines. H1 and H2 shared similar promoter sequences, while H3–H5 had ∼150 bp and 4 bp insertion in the promoter region (Figure 2). However, previous research found no insertion in the *GmSALT3* promoter region (Patil et al., 2016). This difference might due to (1) the ∼150 bp insertion sequence having low similarity with the W82 reference genome, thus it could not be perfectly assembled to the reference genome, or (2) the limitations of next-generation sequencing data in finding long fragment insertions/deletions using paired-end reads of 45 bp or 75 bp (Lam et al., 2010). The 4 bp insertion in the promoter of *GmSALT3* was observed in our 279 resequencing dataset, while the ∼150 bp insertion was not (Figure 1). Reduced gene expression of the salt sensitive haplotypes has been reported by several researchers (Guan et al., 2014b; Do et al., 2016; Lee et al., 2018). This common variation in the promoter region of salt sensitive haplotypes raises the possibility that these insertions might affect the expression of *GmSALT3*.

Patil et al. (2016) conducted six KASPar assays with three structural variants in *GmSALT3* and achieved precise identification of tolerant and sensitive genotypes with over 91% accuracy. Additionally, Lee et al. (2018) developed five molecular markers for the seven haplotype groups in 216 soybean accessions, including three codominant and two dominant markers, and achieved 98.8% predictive accuracy for 173 soybean accessions. In this study, we successfully predicted salt tolerant vs. sensitive accessions using only two of the five codominant markers we developed, i.e., using Pro-Ins to distinguish H1 and H2 from H3 to H5, and using H2-Ins to distinguished H1 from H2. Prediction efficacy was 98.9% for salt tolerant and 100% for salt sensitive accessions.

Interestingly, two accessions (SW10710 and CW10948) with salt-sensitive genotypes showed high or moderate salt tolerance (Lee et al., 2018). We also found that two of our H1 type soybean lines showed salt sensitivity, indicating that other genes may impact salt tolerance in these accessions, and these genotypes are useful material to identify new sources of salt tolerance. Candidate loci that might influence this are hinted at through past literature. For instance, in the population derived from Kefeng No. 1 and Nannong1138-2, a major QTL on linkage group G (Chr. 18) was identified in both green house and field conditions which correlates to altered salt sensitivity (Chen et al., 2008). Furthermore, a novel locus on Chr. 13 related to leaf sodium content was observed in soybean germplasm Fiskeby III (Do et al., 2018). These results suggest that other loci, in addition to the major gene *GmSALT3* affect salt tolerance in soybean. Future work should focus on defining the genetic relationship of these loci and *GmSALT3*; how might alleles be interacting to impact salt tolerance in particular soybean germplasms?

Soybean [*Glycine max* (L.) Merr.] is an essential crop worldwide due to its high protein and oil content; it provides valuable source of nutrition for human consumption and main protein source in for livestock feed (Bellaloui et al., 2014). As one of the major crops grown in China, soybean is widely used for edible oil and soy-based foods such as tofu, soymilk, sauces, and many other products. However, from 2004 to 2016, the land area in Chinese soy production decreased by 25%, much of that land being converted to cropland for maize production (Ren et al., 2021). Reclamation of slightly saline soil, therefore, could be a potential way to increase productivity of soybean yield.

In China, modern soybean breeding started as early as 1923 and regional trials of soybean production began in the 1950s. We found that in soybean landraces, H1 (salt tolerant haplotype) was at highest frequencies in both the NR and HHR, whereas H4 and H5 (salt-sensitive) were highest in the SR. In soybean cultivars, the highest frequencies of H1 were observed in the HHR, and salt-sensitive haplotypes H2 and H5 most frequently occurred in the NR and SR, respectively (Figure 3). In modern breeding lines, the frequency of H2 in the NR increased to 73.3%, and H1 in the SR increased to 41.3%, while H3 and H4 were nearly eliminated from modern breeding lines. The high frequency of the H1 haplotype observed in the HHR is consistent with the distribution of saline soils along the eastern coast of China and saline conditions in the Yellow River Delta (Meng et al., 2016). No significant difference of yield-related traits of tolerant (T) and sensitive (S) near isogenic lines NIL-T and NIL-S has been observed under non-saline field conditions, indicating that the H1 haplotype has no yield penalty (Liu et al., 2016). Additionally, selection of the salt sensitive haplotype H2 in the NR during modern times might be attributed to climate change, water reduction and subsequent decreasing soil salinization in the region (Guo et al., 2016). Likewise, the slight frequency increase of H1 haplotype in the SR (Figure 4) may be related to increasing soil salinization along the Yangtze River Basin due to vegetable production practices (Zhang et al., 2021). That being said, soybean breeding efforts are geared toward many factors including high seed yield, high oil content, and high protein content. Therefore, we cannot rule out the possibility that pleiotropy of *GmSALT3* or linkage drag affects the selection of haplotypes in different ecoregions.

Previously, simple sequence repeat (SSR) markers were used to trace the origin of the salt tolerance gene; however, the multi-allelic nature of SSR markers decreased prediction accuracy of salt-tolerant genotypes (Lee et al., 2004). We were able, however, to clearly distinguish the possible sources of alleles in a soybean pedigree by using functional markers (Figure 6). In addition, previous field studies suggested that the parent strains of Wenfeng 7, Jüxuan 23, and Qihuang 1 exhibited high or moderate salt tolerance (Shao, 1988). In contrast, our research indicated that the parent strain Qihuang 1 was an H5 type salt-sensitive cultivar, thus indicating the predictive value of functional markers and their potential use in soybean breeding.

The ability to select salt-tolerant soybean germplasm via accurate and economic genotyping methods is essential for modern soybean production. Here, we developed PCR-based functional markers of *GmSALT3*, and demonstrated their high efficiency in the selection of salt tolerant or sensitive breeding lines. By using only Pro-Ins and H2-Ins, for which amplicons can be separated on standard agarose gels, salt-tolerant genotypes (H1) are readily distinguished from salt-sensitive genotypes (H2–H5). Most soybean breeding laboratories should be able to employ this method of identification and selection. Our exploration of variation in *GmSALT3* throughout known Chinese soybean breeds, in addition, provided valuable insight into the long-term effect of phenotypic selection on the distribution of *GmSALT3* haplotypes. Further, it provided a basis for the utilization of *GmSALT3* allele ecoregion-specific breeding; which, under changing global conditions, remains key to economic and food security.

## Data Availability Statement

The datasets presented in this study can be found in online repositories. The names of the repository/repositories and accession number(s) can be found in the article/Supplementary Material.

## Author Contributions

RG, RC, and LQ designed the experiments. RG and LQ directed the project. RG performed the design of molecular markers. LY and XL performed the phenotype screen and data analysis. XL and ML performed the genotype test of the soybeans. RG, LY, MG, and LQ wrote the manuscript. All authors commented on the manuscript.

## Conflict of Interest

The authors declare that the research was conducted in the absence of any commercial or financial relationships that could be construed as a potential conflict of interest.

## Publisher’s Note

All claims expressed in this article are solely those of the authors and do not necessarily represent those of their affiliated organizations, or those of the publisher, the editors and the reviewers. Any product that may be evaluated in this article, or claim that may be made by its manufacturer, is not guaranteed or endorsed by the publisher.

## Footnotes

1
https://phytozome.jgi.doe.gov/

## Supplementary Material

Supplementary Figure 1 Geographical distribution of five haplotypes (H1–H5) of *GmSALT3* in Chinese soybean landraces. NR, the northern region of China; HHR, the Huanghuai region of China; SR the southern region of China.

Supplementary Figure 2 Distribution of Chinese soybean landraces possessing haplotype H5-1 and H5-2. Red dots, H5-1; Black dots, H5-2.
