# Supplementary figures and images for: Selection of the Salt Tolerance Gene GmSALT3 During Six Decades of Soybean Breeding in China
Source: Front Plant Sci. 2021 Nov 16;12:794241. doi: 10.3389/fpls.2021.794241 (PMC8635242; doi:10.3389/fpls.2021.794241)

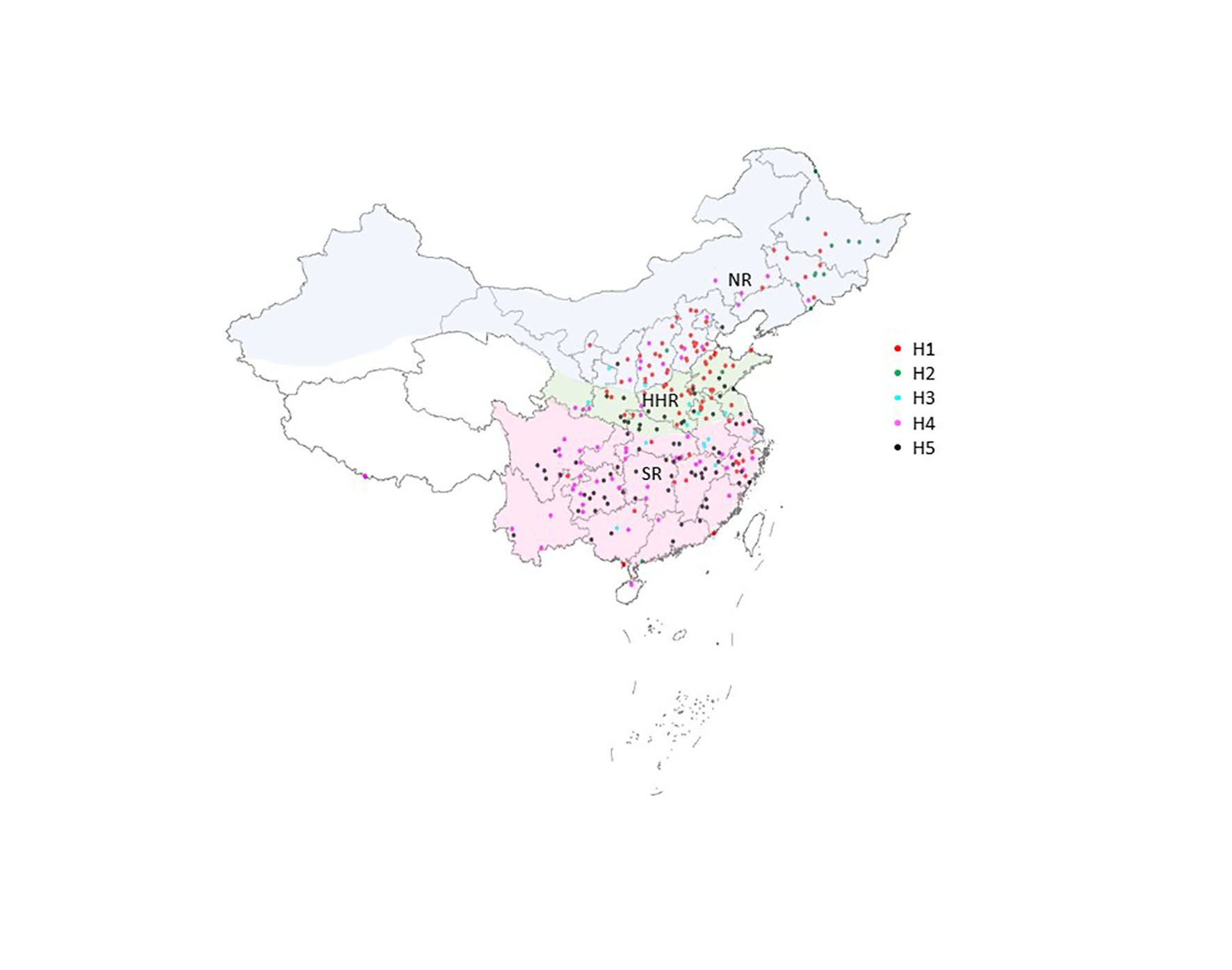

Supplement: Supplementary Figure 1 — Geographical distribution of five haplotypes (H1–H5) of GmSALT3 in Chinese soybean landraces. NR, the northern region of China; HHR, the Huanghuai region of China; SR the southern region of China. [file Image_1.JPEG]

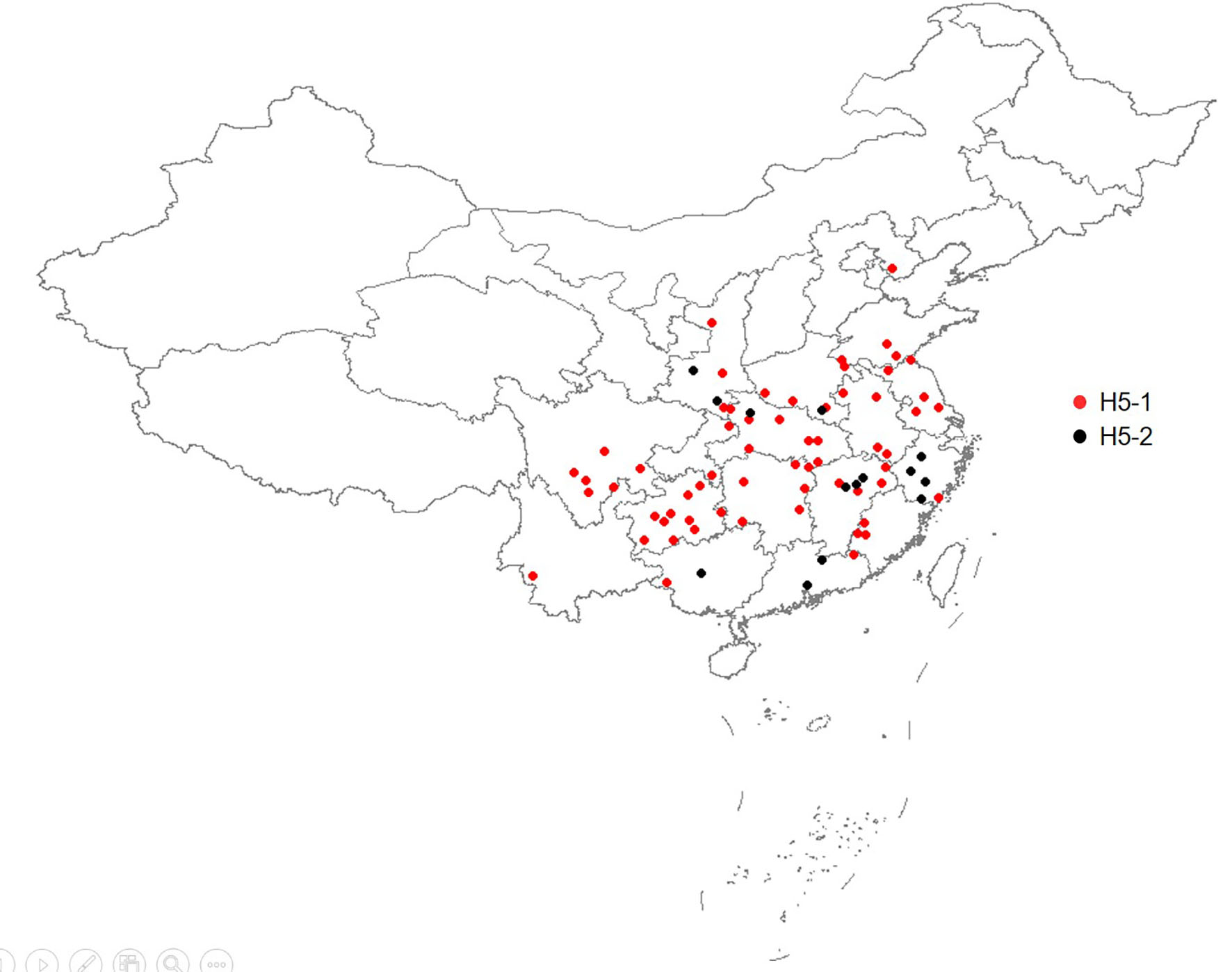

Supplement: Supplementary Figure 2 — Distribution of Chinese soybean landraces possessing haplotype H5-1 and H5-2. Red dots, H5-1; Black dots, H5-2. [file Image_2.JPEG]
